# Mitochondria in disease: changes in shapes and dynamics

**DOI:** 10.1016/j.tibs.2024.01.011

**Published:** 2024-02-23

**Authors:** Brenita C. Jenkins, Kit Neikirk, Prasanna Katti, Steven M. Claypool, Annet Kirabo, Melanie R. McReynolds, Antentor Hinton

**Affiliations:** 1 Department of Biochemistry and Molecular Biology, The Huck Institute of the Life Sciences, Pennsylvania State University, State College, PA 16801, USA; 2 Department of Molecular Physiology and Biophysics, Vanderbilt University, Nashville, TN 37232, USA; 3 National Heart, Lung and Blood Institute, National Institutes of Health, 9000 Rockville Pike, Bethesda, MD 20892, USA; 4 Department of Physiology, Mitochondrial Phospholipid Research Center, Department of Genetic Medicine, Johns Hopkins University School of Medicine, Baltimore, MD 21205, USA; 5 Department of Medicine, Vanderbilt University Medical Center, Nashville, TN 37232, USA; 6 Vanderbilt Center for Immunobiology, Vanderbilt University Medical Center, Nashville, TN 37232, USA; 7 Vanderbilt Institute for Infection, Immunology and Inflammation, Vanderbilt University Medical Center, Nashville, TN 37232, USA; 8 Vanderbilt Institute for Global Health, Vanderbilt University Medical Center, Nashville, TN 37232, USA; 9 Joint first authors

## Abstract

Mitochondrial structure often determines the function of these highly dynamic, multifunctional, eukaryotic organelles, which are essential for maintaining cellular health. The dynamic nature of mitochondria is apparent in descriptions of different mitochondrial shapes [e.g., donuts, megamitochondria (MGs), and nanotunnels] and crista dynamics. This review explores the significance of dynamic alterations in mitochondrial morphology and regulators of mitochondrial and cristae shape. We focus on studies across tissue types and also describe new microscopy techniques for detecting mitochondrial morphologies both *in vivo* and *in vitro* that can improve understanding of mitochondrial structure. We highlight the potential therapeutic benefits of regulating mitochondrial morphology and discuss prospective avenues to restore mitochondrial bioenergetics to manage diseases related to mitochondrial dysfunction.

## Mitochondria are not kidney beans

**Mitochondria** (see Glossary) were discovered in the 1800s [1], and the advent of high-resolution electron microscopy (EM) techniques [2] allowed Palade to identify their unique double membrane in the 1950s [3,4]. Additional studies throughout the mid-1900s [1] revealed the link between mitochondrial structure and function [5], and continual EM advancements have enabled 3D renderings of cellular ultrastructure [6]. Across the 1990s, developments in plastic section electron tomography (ET), which remains clinically relevant today, allowed researchers to explicate the complexity of mitochondrial internal micro-compartments, known as **cristae** [7]. Today, cryo-ET, using principles of rapid freezing, allows for 3D reconstruction of mitochondria and localizing associated large protein complexes (e.g., **ATP** synthase [8]) in their native state, further elucidating the mitochondrial structure–function interplay [9].

**Figure 1.**
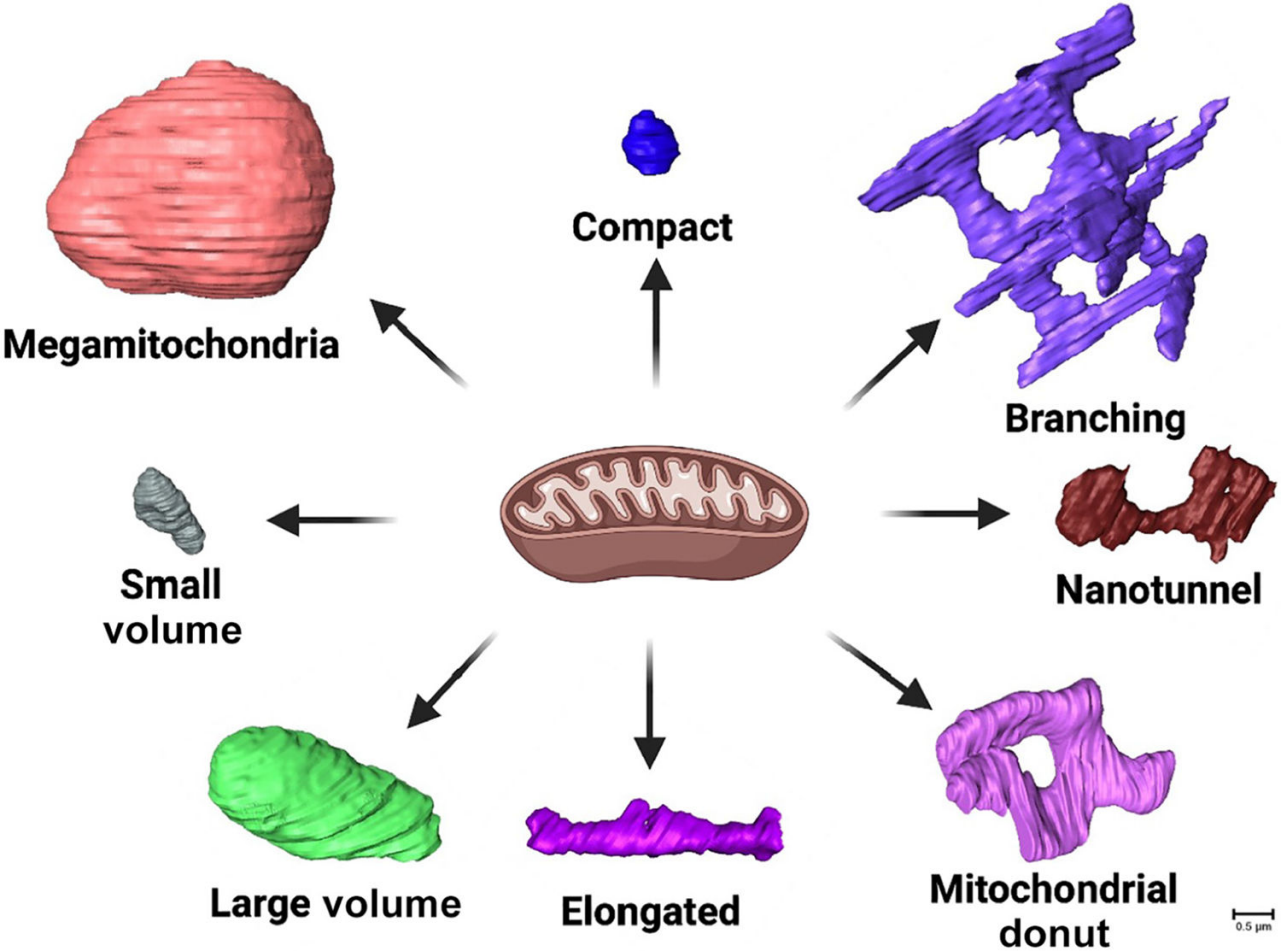
3D structures of mitochondria. 3D structures of mitochondria beyond the kidney bean shape, which may have altered cristae dynamics. The eight so-far observed mitochondrial shapes are compact, branching, nanotunnels, mitochondrial donut, elongated, large volume, small volume, and megamitochondria, and these structures, as obtained from 3D reconstructions from serial block face scanning electron microscopy, may have functional implications.

Recent advances in research have revealed mitochondria as dynamic organelles with heterogeneous shapes and networks both within and among cell types and tissues (Figure 1). For example, mitochondrial morphology can range from a single compact (e.g., in cardiac tissue [10]) or swollen (e.g., in brown adipose tissue [11]) structure to highly branched networks (as in skeletal muscle [12-14]) [15]. Phenotypes can exist ubiquitously across tissue types, although certain tissue types or disease states are more likely to display certain morphologies: for example, liver mitochondria shift between spherical and donut- or c-shaped forms [16,17], cardiac muscle cells have elongated mitochondria arranged in parallel [18], and skeletal muscles have highly connected mitochondrial networks [19]. These phenotypes are best analyzed via high-resolution 3D technology. For instance, low-resolution techniques may mistake nanotunnels for fragmentation [20] and utilizing EM thin sections alone may falsely show cup- or stomatocyte-shaped mitochondria as mitochondrial donuts [21]. Mitochondria can also display distinct morphologies and biochemical properties within a given cell [12,22]: populations near the plasma membrane play essential roles in the functional coupling of ATP-guided ion channels [23], whereas perinuclear networks regulate gene transcription under hypoxic conditions by increasing reactive oxygen species (ROS) in the nucleus [24]. The variation in structure between these subpopulations may reflect differences in function [13,25]. Different intra- and intertissue mitochondrial networks and populations also display different responses to substrates, inhibitors, and oxidative stress, highlighting their complexity, diversity, and plasticity [18,22,26,27].

In addition to producing energy in the form of ATP, mitochondria are involved in calcium signaling, apoptosis, and the oxidative stress response [15,28]. Mitochondrial dynamics and heterogeneity are crucial for regulating these processes and maintaining cellular homeostasis both within individual cells and across an organism [12,29]. Several mechanisms, including mitochondrial biogenesis, transport, **mitophagy**, fusion, and fission, regulate mitochondrial structure and function [29,30]. Other cellular components, such as **lipid droplets** and the **endoplasmic reticulum (ER)**, can also influence mitochondrial structure through interorganellar contact sites that modulate mitochondrial biochemical properties, functional capacity, and dynamics [19,31-33].

Studies of 3D mitochondrial structures indicate that structural changes may occur during aging and that these changes may be associated with functional deficiencies and the development of mitochondria-dependent diseases [10,14]. Additionally, a recent review summarized how mitochondrial phenotypic frequencies may change in response to stimuli [15]. As we explore here, the differential responses to aging among disparate tissue types, which can include the presence of unique mitochondrial structures [34], may be a key pathological factor. However, the relative frequencies of these unique shapes, including MGs, nanotunnels, donuts, and elongated, branched, and compact morphologies (Table 1), must be further elucidated in disease states.

Here, we first describe mitochondrial donuts, MGs, and tunnels in different cell types and in response to different stimuli before discussing how these shapes are related to the organization and orientation of their cristae. We then build on the overview of these internal and overall structures by describing how fusion and fission events, contacts with other organelles, and other structural regulators impact mitochondrial shape. Finally, we explore the potential clinical implications of improved knowledge of mitochondrial structures and dynamics.

## Mitochondrial shapes

### Mitochondria can be donuts

Ahmad *et al.* reported that donut-shaped mitochondria may be an early cellular stress marker [35]. They showed that ROS production induced by complex I and III inhibitors resulted in the transient and reversible transition from tubular to donut-shaped mitochondrial structures, which are associated with increased mitochondrial ROS levels [35]. The formation of these structures may also exhibit tissue dependency. Bleck *et al.* observed in healthy adult mice that, compared with oxidative muscle fibers, glycolytic muscle fibers showed a higher frequency of donut-shaped mitochondria [19]. In contrast, donut-shaped mitochondria may arise more frequently in the liver due to metabolic stress [17]. Donut-shaped mitochondria can also appear in disease states: for example, the memory impairment characteristic of Alzheimer’s disease occurs concomitantly with an increased prevalence of neuronal donut-shaped mitochondria [36].

Our understanding of donut-shaped mitochondria has evolved alongside the techniques that allow us to model them, including EM [36] and live-cell imaging [37]. Donut-shaped mitochondria form when the opposite ends of a mitochondrion fuse [37], although the mechanism underlying this process remains largely unknown. While it has been suggested that donut-shaped mitochondria can form independent from fission, these fission-independent models likely show collapsed mitochondria that phenotypically appear as cup shaped, which represent a fission-independent response to mitochondrial membrane potential loss [21]. Donut mitochondria have been observed under hypoxic conditions [37], and are associated with reduced mitochondrial fusion events and changes in osmotic pressure that lead to diminished neuronal synapses [36,38]. When returned to nonstressed conditions, donut-shaped mitochondria readily revert to other morphologies [37], suggesting that the donut shape may represent an adaptive response for increasing mitochondrial surface area for organelle contacts [15] or resisting mitophagy [39]. Donut-shaped mitochondria maintain the overall mitochondrial membrane potential (generated by an electrochemical gradient across the inner mitochondrial membrane). This suggests that donut formation may protect against mitochondrial function loss by reducing the Gibbs free energy of the system, which inversely increases membrane potential [38,39]. However, it is unclear if other mitochondrial structures depend on Gibbs free energy, as well as associated thermodynamic temperature. While past results have found that even under uncouplers, mitochondria donut diameters tend to be approximately 1.3 μm [37], it is unclear if donut size may determine the bending energy, a major barrier to donut formation that may be counterbalanced by stress-inducible mitochondrial osmotic pressure [38]. Furthermore, as the study of mitochondrial donuts continues, advanced methods must be utilized, as fluorescence and thin EM techniques may mistakenly identify discoid-, curved-, and vase-shaped mitochondria as donuts [21].

### Mitochondria can be mega

Mitochondria that swell to two to three times their typical size (from an average length of ~5 μm up to 10 μm) while maintaining outer membrane integrity are characterized as MGs [40]. Although these phenotypes can reach the size of the nucleus [41], ultrastructural or 3D reconstruction techniques are useful to identify them [40,41]. MGs form in response to diseases, such as nonalcoholic fatty liver disease [41], and environmental or cellular stressors [40]. However, the effect of MG formation on cell function is unknown.

The mechanism underlying MG formation is not fully understood, but mitochondrial fission and fusion defects are likely contributors. During stress, fusion increases mitochondrial complementation and maximizes oxidative capacity as a compensatory mechanism [42]. MG formation may also result from stress-induced hyperfusion [43], the accumulation of damaged or dysfunctional mitochondria due to fission or fusion defects [44], and mitochondrial membrane potential–dependent changes in mitochondrial fission and fusion [45].

The **ER-associated degradation (ERAD) pathway** also regulates mitochondrial size, and ERAD deficiency leads to MG formation, suggesting that MG formation may be regulated through inter-organelle mechanisms [46]. Given there is a known association between ER stress and mitochondrial elongation [47], Zhou *et al.* investigated how ERAD deficiency affects mitochondria and found that deficiency in the ERAD component Sel1L promotes MG formation in brown adipocytes, possibly due to inhibited fission or accelerated fusion [46]. This finding suggests that MG formation may be affected by changes in expression of specific proteins at the **mitochondrial–ER contact sites (MERCs)**.

### Mitochondria can form tunnels

Investigations of several diseases have uncovered various specialized mitochondrial shapes. In Alzheimer’s disease, 3D EM revealed a novel elongated mitochondrial phenotype known as ‘mitochondria-on-a-string’ [20] – later termed nanotunnels. As reviewed [48], mitochondrial nanotunnels are thin (<200-nm diameter) double membranes that connect adjacent mitochondria (up to 30 μm in distance) and can transport proteins and molecules across the mitochondrial membrane into connected organelles. Nanotunnels have been detected in diseased tissue and are hypothesized to result from incomplete fission [48], although it is unclear whether their formation requires specific types of fission (e.g., midpoint or endpoint). The effect of nanotunnel formation on the distribution of dynamic mitochondrial proteins and the structure dependence of protein localization also requires investigation. The spatial orientation of mitochondrial proteins may reveal nanotunnel formation mechanisms, although the specific pathways involved in healthy and disease conditions remain underexplored.

In human skeletal tissue samples from a patient with mitochondrial disease and large-scale mitochondrial DNA (mtDNA) deletion, Vincent and colleagues identified an 18.1-fold increase in nanotunnel formation compared with healthy samples [13], suggesting a correlation between nanotunnel formation and mtDNA mutations, although other mechanisms may be involved. Mitochondrial fragmentation due to excessive fission indicates cell damage [42], and nanotunnels may form in response to cell damage caused by mtDNA mutations. There are other examples of proteins involved in nanotunnel formation: **ryanodine receptor 2 (RYR_2_**)-dependent calcium homeostasis is known to regulate nanotunnel formation [49], and **activating transcription factor 4 (ATF4)** and other ER stress proteins could play a role in this regulation [50]; little else is known about these regulatory mechanisms and would benefit from further exploration.

One potential complication of studying nanotunnels is that they are among the mitochondrial shapes that arise under hypoxic conditions [48] and therefore may result from improper, hypoxia-inducing fixation techniques [51]. Thus, the standardization of fixation and quantification techniques must be improved to ensure that mitochondrial structures represent altered metabolic conditions rather than improper fixation [52].

## Connecting mitochondrial shape to cristae dynamics and membrane potential

**Figure 2.**
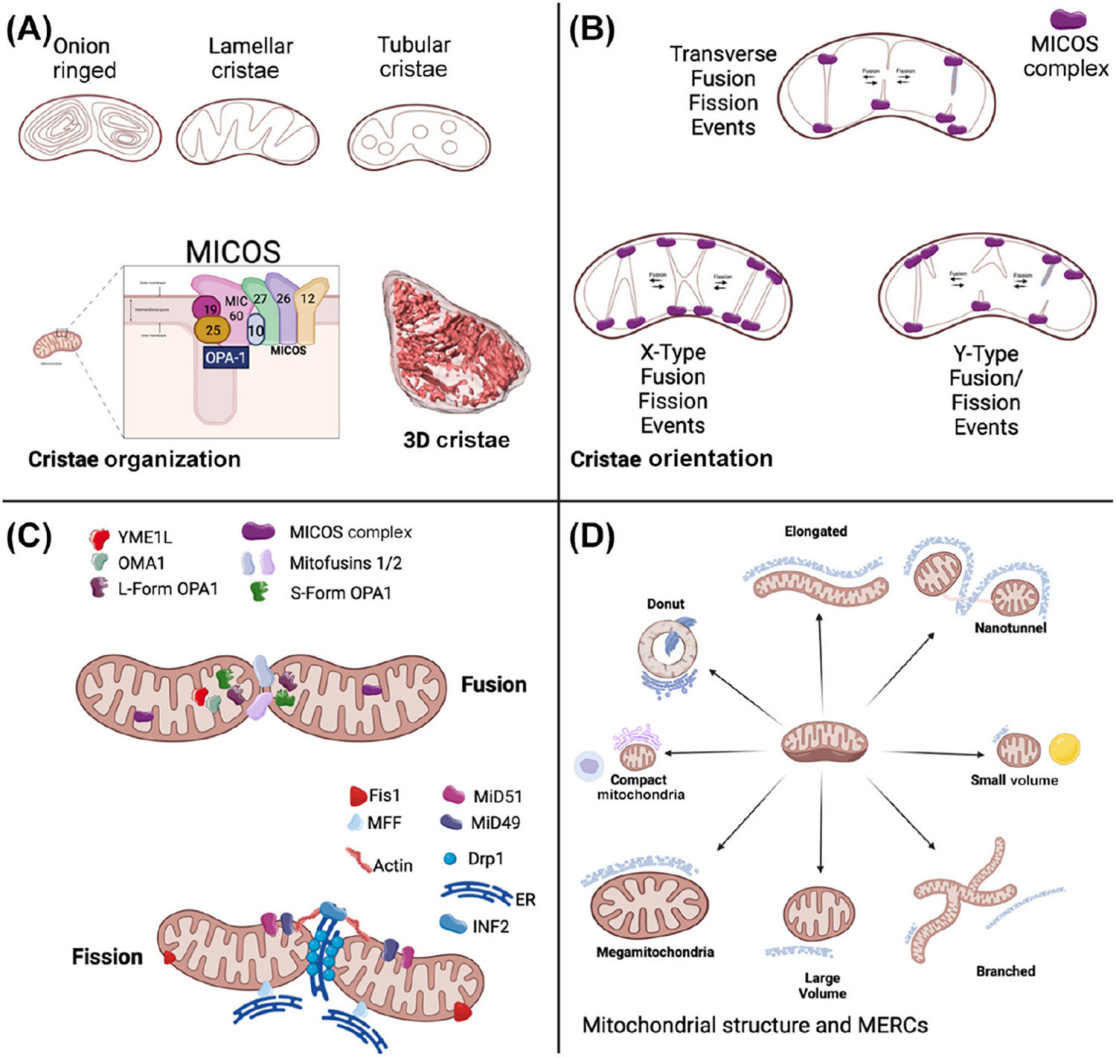
Current state of mitochondrial dynamics beyond their phenotype. (A) Mitochondrial cristae can take on several morphologies, potentially due to the mitochondrial contact site and cristae organizing system (MICOS) complex, and may be imaged in 3D. (B) Mitochondrial cristae can form x- and y-type fusion and fission events, indicating cristae-specific dynamics. (C) Known factors involved in mitochondrial dynamics and potential new regulators. Fission can be mitochondrial fission factor (MFF)- or mitochondrial fission 1 protein (FIS1)-mediated, but the roles of these regulators and others, such as YME1L, in regulating 3D mitochondrial shape remain unclear. (D) Contact sites have several key phenotypes, which may depend on mitochondrial shape. Abbreviations: MERCs, mitochondria–endoplasmic reticulum contact sites; OPA1, optic atrophy 1.

The diversity and heterogeneity of mitochondrial shapes, including those described in the preceding text, affect the spatial orientation of cristae and are modulated by the **mitochondrial contact site and cristae organizing system (MICOS) complex** [53], as well as mitochondrial signaling pathways (Figure 2A). Although cristae have unique dynamics independent of mitochondrial structures, common fusion proteins, including **optic atrophy 1 (OPA1)**, which is epistatic to the MICOS complex [54], and **YME1L**, regulate their dynamics through crista junction closing and opening [55]. Cristae are tubular or lamellar under normal conditions [56]; abnormal shapes, such as spherical [55] and onion-ring cristae [57] (Figure 2A), arise in abnormal conditions, such as mitochondrial dysfunction and mitochondrial disorder, respectively. These abnormal shapes may optimize **intracristal spaces**, but their functional implications remain understudied [57]. In some mitochondrial phenotypes, such as MGs, a paucity or abnormality of cristae is common [41,46]. However, while the cristae structural differences may confer functional changes [15], the interdependence between cristae shape and mitochondrial phenotype remains poorly explicated.

Cristae harbor the machinery for oxidative phosphorylation and ATP production [56], and their organization is intricately linked to energy production efficiency and overall mitochondrial health [54,58]. Advanced imaging techniques, such as 3D **MINFLUX** nanoscopy, have revealed the complex organization of cristae and the MICOS complex [53], which is involved in structural cristae remodeling [59]. Cristae organization disruptions and alterations are implicated in neuro-degenerative, metabolic, and cardiovascular diseases [60,61].

Notably, the interplay between crista shapes and the **mitochondrial permeability transition (mPT)** must be further explored. Though the molecular mechanism is unknown, elevated calcium may open an mPT pore to an unspecified channel, resulting in ruptured cristae and eventual cell death [62]. As mPT pores are regulated by matrix cyclophilin D, they may consist of ATP synthase dimers [62]; however, this suggestion remains controversial [63]. Beyond the role of mPT in disease [64], the mPT has clear roles in modulating membrane potential [62]. This offers implications in mPT regulating membrane potential-dependent mitochondrial structure, such as the formation of cup-shaped cristae upon loss of membrane potential [21]. Because individual cristae can have distinct membrane potentials [59], and because ATP synthase dimerization regulates crista morphology [65], the interplay between mPT, ATP synthase dimerization, cristae structure, and mitochondrial structure must be explicated, possibly through cryo-ET, which enables visualization of ATP synthases [8].

Cristae undergo ongoing and dynamic membrane remodeling events, which may be implicated in mPT pore formation or shape tendency. In these continuous cycles, similar to OPA1-mediated cristae junction flux [55], cristae are continuously remodeled by **transverse, x-type, y-type**, and other types of cristae fusion and fission (Figure 2B) [60,66]. However, this process remains poorly elucidated. For example, crista dynamics may be determined by the spatial orientation of the mitochondrial MICOS complex, which may depend on the mitochondrial shape. Yet, it is unclear whether the relative rates of types of crista remodeling events affect their membrane potential, tendency toward crista organizational types, or mitochondrial shape, especially in the case of pathological conditions.

## New players in the regulation of mitochondrial shape

### Fusion and fission

Discussions of mitochondrial dynamics often include a common set of core proteins, including OPA1, dynamin-related protein 1, mitofusin 1, and mitofusin 2. However, the current understanding has evolved beyond these factors (Figure 2C). In addition to its role in cristae remodeling [67], OPA1, when in its long, active form, plays an important role in fusion. Upstream, OPA1 can be inhibited through cleavage by YME1L and **OMA1** [68], resident proteases that modulate mitochondrial dynamics. Similarly, actin constricts to enact fission, and other mitochondrial dynamics proteins, including MiD49 and MiD51, also play a role in fission [69]. Notably, actin constriction prefission is orchestrated by ER-associated IFN2 [70], highlighting the interconnectedness of MERCs and mitochondrial fusion–fission dynamics.

Although past studies have suggested that mitochondrial networks are determined entirely by fusion and fission dynamics [30], the involvement of various fission factors, such as **mitochondrial fission factor (MFF)** and **mitochondrial fission 1 protein (FIS1)** [71], in determining mitochondrial phenotype remains unclear. Kleele *et al.* demonstrated that distinct forms of fission occur based on spatial orientation of FIS1 and MFF [71], suggesting that protein distribution may contribute to mitochondrial morphology. A recent study found that FIS1 overexpression results in increased donut formation [72], but whether MFF overexpression has similar effects has not yet been explored. Although previous studies have shown that mitochondrial fission events are integral to mitochondrial morphology outcomes [29], the specific contributions of unique 3D phenotypes, such as MGs, and contact sites require further exploration.

### Sex-dependent modulators

Estrogen, which declines with aging, is associated with changes in mitochondrial morphology. In ovariectomized monkeys, donut-shaped mitochondria are a pathological indicator of age-related Alzheimer’s disease, and normal mitochondrial structure and function can be rescued by estrogen treatment [36]. Notably, an *in vivo* mouse study showed that 17β-estradiol (E2) in mitochondrial membranes decreases membrane viscosity, which can have adverse effects given that optimal **membrane fluidity** is necessary for proper mitochondrial function [73]. Mitochondrial membrane potential changes may occur concomitantly with alterations in membrane fluidity [74], and mitochondrial cristae have functionally independent membrane potentials [59]. Therefore, it could be informative to examine whether E2 inner-membrane localization occurs, and, if so, whether such localization is cristae-dependent or E2 binds to specific areas of the inner mitochondrial membrane. Furthermore, sex-dependent differences in mitochondrial structure across aging remain unclear, which has implications for uncovering if estrogen may modulate mitochondrial structure and confer a protective effect.

### Nucleoids

**Nucleoids** contain mtDNA, which encodes several proteins and RNAs (tRNA and r**RNA**) involved in protein synthesis, mechanistically enabling submitochondrial organization and maintenance of mtDNA [75]. As reviewed [76], nucleoids, dictated by tissue type and age, often have integral roles in modulating metabolism. Lewis *et al.* convincingly demonstrated that mtDNA signaling involves MERCs [77]. Nucleoid transportation at MERCs is further determined by the MICOS complex [78], suggesting the potential involvement of cristae. Although fusion and fission are understood to contribute to mitochondrial genome integrity through the maintenance of nucleoids distributed to daughter mitochondria [79], it is unclear how different mitochondrial shapes affect nucleoid replication and distribution. For example, the large size of MGs may affect MERC formation [46] and consequently impact nucleoid distribution. The mechanisms underlying the formation of different mitochondrial shapes may also impact the number and distribution of nucleoids; for example, if MGs are formed from multiple fusion events [41], they may contain more than one nucleoid, which may impact their function [76]. Thus, further study is needed to understand how changes in mitochondrial structure affect mtDNA and vice versa. Utilizing new techniques, such as Mitomate tracker [80], for the automated quantification of nucleoid distribution in tissues where mitochondrial phenotypes are naturally found (e.g., liver cells for MG [41]), may offer greater insight into this topic.

### MICOS complex

The MICOS complex is involved in aging [10,14,81] and, as described in the preceding text, cristae organization (Figure 2A) [66], and may also affect overall mitochondrial shape [10,14]. As previously reviewed [60], the MICOS complex contains multiple proteins, although their independent roles and necessity for MICOS complex functionality remain unknown. One such protein, mitofilin, is important in nucleoid organization and metabolism [82], suggesting that nucleoid distribution may involve the MICOS complex or that its various components play essential roles beyond metabolism. The MICOS complex also interacts with the sorting and assembly machinery complex [83], but the necessity of these two complexes for the formation of specific mitochondrial shapes remains unclear.

Tissue-dependent deletion of the MICOS complex and its specific proteins may advance our understanding of how the MICOS complex affects the gross mitochondrial structure and cristae spacing. Tools have recently been developed to enact overexpression of MICOS complex components [84]. However, floxed models for the deletion of MICOS complex components are lacking, and it remains unclear whether the MICOS complex subunits can be deleted in a tissue-specific manner to better identify the specific functions of individual components.

### Other potential modulators

As this section highlighted, our understanding of mitochondrial modulators must continue to evolve. One promising avenue is the study of mitochondrial transporters, specialized proteins that mediate the movement of essential small molecules that are critical in maintaining cellular energy production, metabolism, and redox balance (Box 1). Mitochondrial processes involving the internal diffusion of metabolites and ions are influenced both by the shape of the inner membrane, in turn, affected by crista dynamics, and transport across the membrane, making transporters novel potential upstream regulators of mitochondrial structure. Understanding their integrated functions in mitochondrial biology may lead to the identification of therapeutic targets and novel modulators of mitochondrial structure in various diseases.

## Mitochondrial shapes are influenced by their organellar connectome

Mitochondrial structures should be considered in the context of the entire organellar connectome, including MERCs and contacts with lipid droplets and autophagic materials [31]. As noted previously, recent findings have shown that MERCs have regulatory roles in both fission [32] and fusion [33], thereby modulating mitochondrial morphology. However, it is unclear whether different types of contact sites with unique biochemical roles exist. For example, centers of donut-shaped mitochondria are often filled with sarcoplasmic reticulum (SR), ER, or lipid droplet contact sites (Figure 2D) [19]. Tangentially, mitochondria form contacts with lipid droplets to perform fatty acid oxidation [85]. Studies of these lipid droplets show that their mitochondrial contact sites can either be anchored or formed in ‘kiss-and-run’ interactions, each of which has distinct functional implications and formation pathways [85,86]. As organelle recruitment may differ across distinct structures, future studies should examine how different mitochondrial shapes affect MERCs.

Beyond MERCs, deeper investigation is needed into how mitochondrial interactions with other organelles, such as recycling machinery [87], may dictate morphology. Notably, artificial transgene tethers have arisen as a potential therapy to regulate MERC formation, which may thereby alter mitochondrial morphology [32,33,88]. This application represents a potentially valuable opportunity to reduce cardiac modeling [88]. However, no transgenes have been identified as potential tethers for other mitochondrial contact sites.

MERC formation may facilitate the interorganellar transfer of mtDNA, Ca^2+^, or other ions [89]. Lavorato and colleagues reported that decreased RYR_2_ activity, which is associated with SR Ca^2+^ imbalances, increased nanotunneling without changing fission rates [49]. This phenomenon may be connected to ATF4-mediated pathways, which are associated with mitochondrial Ca^2+^ homeostasis [90]. Thus, nanotunnel formation represents a compensatory cellular response to Ca^2+^ dysregulation, which is further supported by the increased rate of nanotunnel formation in disease states [13,48], suggesting an interplay between impaired MERC formation and the emergence of structures representative of disease states.

The contributions of different crista types and the involvement of mitochondrial proteins, such as the MICOS complex or fission factors, in the formation of contact sites, also remain unclear. However, the current understanding of how MERCs dictate MICOS complex orientation [91] may help explain how MERCs can dictate cristae structure.

## Future clinical considerations of mitochondrial structure

mtDNA changes were first considered to be functionally linked to myopathies in the 1980s [92], and the role of mitochondria in medicine has continued to evolve ever since [93]. However, despite the known association of mitochondrial structure with pathologies [36,41], it still has limited clinical applications, and the functional impacts of different mitochondrial shapes, including their relative frequencies in different disease states and tissues, remain unclear. Certain mitochondrial phenotypes, such as donut mitochondria, may serve as markers of disease [36], and recent studies have noted changes in the prevalence of certain mitochondrial phenotypes in response to specific pathological conditions [13,15,41]. Frequency changes for specific mitochondrial shapes could further indicate cellular stress, inflammation, or metabolic alterations. Notably, mitochondrial mass, stress conditions, and motility dynamically affect fusion and fission rates, resulting in heterogenous micropopulations [94]. Further elucidation of how these factors affect the drivers of fission, such as MFF or FIS1 [71], may support clinical diagnosis.

Different mitochondrial distributions have been established for subsarcolemmal, perinuclear, and intermyofibrillar regions of mammalian cardiac muscle [26,95], but whether these distribution differences affect mitochondrial function has not been determined. Further, various mitochondrial populations have displayed differences in relative energetics [96], and variations in the aging rates of tissues [34] may indicate tissue- [14] or population-dependent mitochondrial regulation mechanisms contributing to pathology. However, many studies have focused on murine models; a comprehensive understanding of mitochondrial structures and functions will require the characterization of mitochondrial phenotypic frequencies across various tissue and cell types from various organisms, including humans, to account for potential tissue-specific effects.

Research must examine the processes and pathways affected by mitochondrial genomes to elucidate how ancestry affects population health studies. The presence of several different alleles within one patient, known as **mtDNA heteroplasmy**, may also contribute to differences in mitochondrial shape as mtDNA may affect both mitochondrial shape and organelle–organelle interactions [77]. However, it is unclear whether mtDNA alterations affect mitochondrial shape across ancestry, race, ethnicity, and geographical order. mtDNA has a high mutation rate, and some mutations are deleterious, predisposing carriers toward certain mitochondrial diseases. Moreover, depletion of mtDNA induces mitochondria fission factors, impacts 3D formation, and changes the actin cytoskeleton [97]. Therefore, identifying female germline mtDNA mutations, learning how these mutations become predominant, and determining the possible pathways impacted are important avenues for understanding mtDNA-related inheritable diseases and interactions with mitochondrial structures.

A program using deep learning processes and large-input, open-source software could determine whether mitochondrial shapes contribute to disease prognosis and associated diagnosis. Future clinical applications will likely require machine learning techniques to render 3D mitochondrial structures. For example, the recently developed MitoEM program has successfully segmented mitochondrial images in both human and mouse tissues, representing a promising avenue in accelerating the speed of mitochondrial reconstruction and subsequent quantification [98]. However, this pipeline has not been tested in clinical applications, and EM technique standardization is still lacking [99]. Moreover, current machine learning techniques may struggle to accurately survey mitochondrial phenotypic frequencies in human biopsy tissue without a dedicated database or atlas. While drastic improvements must be made in EM techniques, not limited to validation, technique standardization, and dedicated databases for accurate phenotypic surveys in human biopsy tissues, as these technologies continue to rapidly improve, clinical applications may be a promising future avenue.

## Concluding remarks

Future studies of 3D mitochondrial structures will be facilitated by developing microscopy techniques [99], such as serial block-face scanning electron microscopy (SEM) [100], correlative light EM [101], transmission EM [99,102], focused ion beam-SEM [103], cryo-ET [9], and mass spectrometry imaging [104] (Box 2). Using these techniques in tandem to define protein distribution and the 3D structures of mitochondria and cristae may clarify how different protein concentrations regulate mitochondrial phenotypes, such as through specific concentrations of proteins in distinct geographies. These methods may be further combined with techniques, such as mitoRACE, that provide information on energy metabolism [22]. Although these techniques are not yet widely used in clinical settings, they may reveal useful information about mitochondrial ultrastructure for clinical contexts.

We believe that accurate quantification of mitochondrial shapes is essential for characterizing their roles in cellular functions and disease, and that we must define parameters for classifying distinct phenotypes, including the distances of nanotunnels and the sizes of donuts and MGs. Importantly, our understanding of mitochondrial structure must consider the potential pluralistic hallmarks of disease states (Figure 3, Key figure). Future research may explore how the presence and frequency of these structures differ between tissues and investigate the mechanisms of abnormal mitochondrial morphology formation in various diseases (see Outstanding questions for additional topics of study). For example, recent studies have shown that the MICOS complex is mechanistically implicated in cardiac dysfunction [81], but the clinical impacts of MICOS complex expression changes in different disease states must be further explored. In conclusion, future study of mitochondrial shape, arrangement, and heterogeneity, in addition to identifying novel regulators, will be crucial to understanding their roles in cellular homeostasis and how they are altered to respond to the physiological state and energy demands of the cell.

## Key figure

### Mitochondrial structural hallmarks of disease

**Figure 3.**
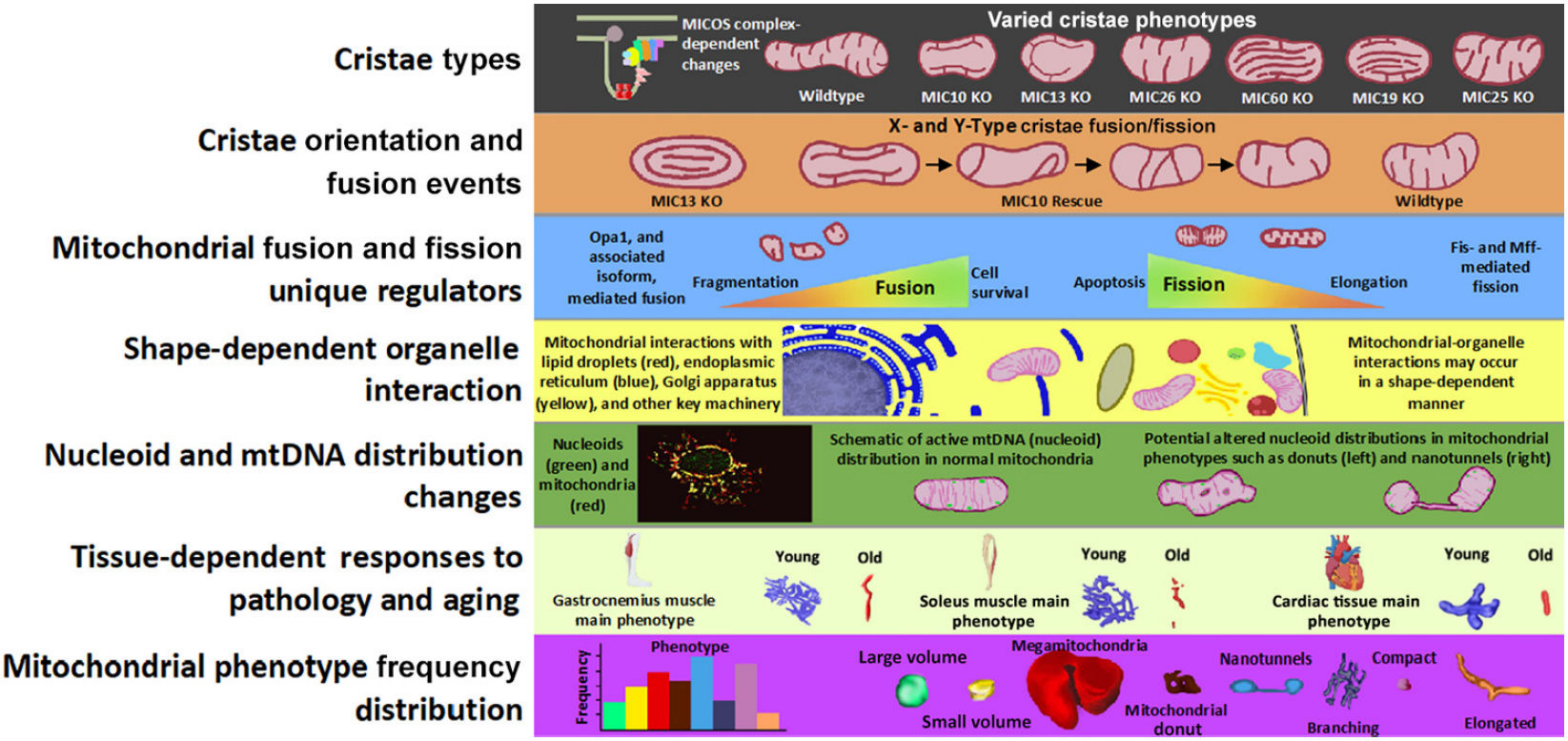
Based on our review, we propose the following essential aspects to consider when researching mitochondrial structures and how their structure may influence their function: cristae types [124]; cristae orientation and fusion/fission events [66,124]; mitochondrial fusion and fission [30,71]; organelle interactions [31]; nucleoid distribution changes [76,77]; tissue-dependent changes [10,11,14,34]; and relative mitochondrial 3D structural phenotypes [15].

## Figures and Tables

**Table 1.** Eight 3D phenotypes of mitochondria

Phenotype	Proposed functional relevance	Potential disease relevance	Refs
Small volume	Potentially occur posterior to fission. Often found in regions of high energy need so they may deliver energy rapidly. Also commonly observed throughout the aging process.	Reduced volume may be representative of Parkinson’s and Alzheimer’s disease.	[10,14,15]
Large volume	Potentially occur posterior to fusion. The larger volume allows for increased energy generation by a few mitochondria. Potentially linked to a loss of membrane potential.	Excessive enlargement may be linked to heart failure.	[11,123]
Compact	Exhibit enhanced oxidative phosphorylation and potential rearrangement of cristae organization.	May be associated with oxidative stress-related conditions, such as ischemia–reperfusion injury and aged heart.	[10,15]
Elongated	Exhibit a higher surface area:volume ratio, potentially for increased interactions.	Excessive elongation may occur in neurodegenerative diseases.	[15]
Megamitochondria	Potentially a sign of mitochondrial swelling or a compensatory mechanism for cellular stress.	May arise in liver diseases, such as alcoholic liver disease and nonalcoholic fatty liver disease.	[40,41,46]
Nanotunnel	Possibly a sign of mitochondrial immobility. Enables ion communication between mitochondria and potential mitochondrial dynamic protein transfers.	Arise in skeletal muscle of mtDNA disease, in cardiomyopathy, and cardiac calcium impairment.	[48,49]
Mitochondria donut	Potentially a sign of incomplete fission or a compensatory mechanism for increasing surface area interactions at the cost of cristae.	Arise in the brain of individuals with cognitive decline.	[17,35-37,72]
Branching	An interconnected network of mitochondria that can enable distribution of energetics in the mitochondria.	Altered branching may be observed in cancers.	[10]

## Highlights

Mitochondrial shape heterogeneity in disease may inform novel therapeutic targets.

3D electron microscopy is developing as a potential clinical diagnostics tool for exploring mitochondrial pathology.

Characterizing mitochondrial structure may elucidate tissue-dependent differences and aid in understanding different cell types and conditions associated with different health outcomes.

## Box 1. Mitochondrial transporters – facilitators of cellular metabolism that may affect structure

Mitochondrial ADP/ATP carriers play crucial roles in cellular energy production by transporting ADP into the mitochondrial matrix for ATP synthesis and exporting ATP to fuel the cell. To meet the inorganic phosphate requirements for cellular oxidative phosphorylation and ATP synthesis, the mitochondrial proton/phosphate symporter transports inorganic phosphate from the cytosol into the mitochondrial matrix [106]. Most mitochondrial transporters belong to the solute carrier family 25 (SLC25), which are typically embedded in the mitochondrial inner membrane and play critical roles in supplying this organelle with essential metabolic intermediates and coenzymes. Recently, three independent research groups discovered that SLC25A51 serves as a mammalian NAD^+^ transporter [107-109], revealing how mitochondria are supplied with this key cofactor that fuels most cellular redox reactions. NAD^+^ functions as both an electron acceptor (in its oxidized form) and donor (in its reduced form) [107-109] and is an important signaling molecule in numerous cellular processes. The identification of SLC25A51/MCART1 as the primary mitochondrial NAD+ transporter represents a significant advancement in understanding cellular metabolism.

Mitochondrial transporters play crucial roles in cellular metabolism and are linked to various diseases, including muscular dystrophy [110]. They may also support drug delivery applications; for example, reduced mitochondrial NAD^+^ levels and protein hyperacetylation result in an anti-tumor effect when combined with aspirin [111]. The impacts of mitochondrial transporters are evident in aging populations: mitochondrial dysfunction, oxidative stress, inflammation, and genomic instability have been proposed as hallmarks of aging [10,112,113]. SLC25 transporters facilitate vital processes, and growing evidence indicates that some SLC25 family members are involved in pathological states, including cancer [114]. Similarly, SLC1A5 was recently found to mediate mitochondrial glutamine metabolism in cancer, indicating that SLC transporters may also modulate levels of ROS [115]. As previously reviewed [116], ROS can affect calcium signaling in mitochondria, suggesting further involvement of MERCs. Cup-shaped and donut mitochondria are suggested to be adaptive mechanisms to respond to osmotic stress and oxidative damage that occurs following the loss of mitochondrial membrane potential [21]. Thus, SLC25 members, including potentially SLC25A51, may modulate mitochondrial structure by promoting osmotic stress. However, it remains unclear whether SLC transporters can also mechanistically shift mitochondrial structures to those emblematic of disease states. More work is needed pointedly focused on testing how transporter function can result in changes in mitochondrial structure, which may uncover potential avenues for intervention.

## Box 2. Emerging technologies to consider for surveying mitochondrial dynamics

2D and 3D imaging may be able to detect unique mitochondrial phenotypes, although this identification requires new standards for mitochondrial characterization. Mitochondrial shape could serve as a cost-effective indicator of disease progression, and 3D imaging may be able to distinguish between healthy and diseased clinical samples.

Current 3D imaging techniques reveal diverse mitochondrial structures, including nanotunnels, megamitochondria, hyperbranching, and fragmentation [52]. For example, focused ion beam (FIB)-scanning electron microscopy (SEM) combines FIB milling with SEM to generate high-resolution 3D reconstructions of subcellular structures [52,103]. FIB-SEM has been used to study mitochondrial morphology, cristae organization, and MERCs in various cell types under different conditions [51,117] and can reveal alterations in mitochondrial structure that clarify the contributions of mitochondrial morphology to disease progression and pathogenesis.

Alternatively, cryo-ET can produce 3D images of the mitochondrial native state [9,118]. Beyond the mitochondrial ultrastructure, the high resolution of cryo-ET can render protein complexes [9], including changes in respiratory chain supercomplexes [119]; ATP synthase [8]; tethers of mitochondrial contact sites, such as those tethering mitoribosomes to the inner mitochondria membrane [120]; and cytoskeletal filament interactions [121]. These cryo-ET-derived nanoscale details provide greater mechanistic insight into the formation of mitochondria shapes and associated contact sites.

Recent advances have led to clinical applications of correlative light electron microscopy (CLEM), a powerful imaging approach that combines the molecular specificity of fluorescence microscopy with the ultrastructural resolution of electron microscopy [122]. CLEM has been used to study real-time changes in mitochondrial protein localization and oligomerization [101]. CLEM allows researchers to visualize specific proteins or organelles within the context of the overall cellular architecture at high resolution, including changes in mitochondrial morphology, enabling a more comprehensive understanding of mitochondrial structure and function [122].

High-resolution mass spectrometry imaging (MSI), another powerful imaging technique, simultaneously visualizes multiple isotopes within a single sample [104], enabling researchers to study the distribution of specific elements within mitochondria as well as potential mitochondrial alterations in disease states. MSI can also be used to investigate various aspects of mitochondrial function, including the distribution of proteins and lipids and the turnover of mitochondrial components. The developing technology Multicolor 3D MINFLUX [53] can assess the spatial orientation of proteins within the mitochondria, such as the MICOS complex, which may have implications for overall activation. Thus, future investigations may uncover how protein distribution affects mitochondrial morphology.

## Outstanding questions

Are altered mitochondrial shapes related to human pathology? Can the regulation of mitochondrial morphology mitigate pathological development?

Are differences in machinery or genome activation across mitochondrial phenotypes related to mtDNA inheritance and mitochondrial function?

Do homotypic (same type) and heterotypic (different types) fusion or fission events govern the formation of distinct mitochondrial shapes?

Does the spatial orientation of the MICOS complex determine mitochondrial shapes?

What are the effects of x-type, y-type, and other types of cristae cycle dynamics?

Does the relative orientation of cristae differ among different mitochondrial shapes?

Do alterations in cristae types and orientations precede changes in mitochondrial shapes?

What roles do cristae play in modulating mitochondrial proteins or related carriers (e.g., SLC25)? Do interactions of SLC25A46 with OPA1 and the MICOS complex alter cristae orientation?

Do tissue-specific differences in the presence and frequency of mitochondrial shapes exist? What mechanisms are involved in establishing abnormal mitochondrial morphologies in various disease states?

Do differences in protein distribution determine 3D phenotypes?

Are the relative frequencies of various mitochondrial shapes associated with positive or negative disease states?

Why are some forms of MERCs associated with specific mitochondrial shapes?

Can artificial intelligence and other techniques be used to detect patterns in mitochondrial shape frequency?

What molecular mechanisms underlie tissue-specific expression and regulation of mitochondrial transporters in various metabolic contexts? Do these mechanisms crosstalk with mitochondrial shapes?

How do variations in mitochondrial shapes impact the localization and activity of mitochondrial transporters?

How do the shape and curvature of the inner mitochondrial membrane influence the distribution and shape of cristae or the organization of mitochondrial transporters?

## Glossary

Activating transcription factor 4 (ATF4): transcription factor that plays a key role in the cellular response to stress, including endoplasmic reticulum stress, and is known to induce the integrated stress response.
ATP: nucleotide produced by mitochondria during cellular respiration that serves as the primary energy currency of cells through its interconversion with ADP.
Cristae: partitions dictated by invaginations and separated from the rest of the inner mitochondrial membrane through cristae junctions. These infoldings increase the inner membrane surface area, allowing for increased ATP synthesis. Cristae are known to take on a multitude of shapes, including onion, tubular, and lamellar. Previously reviewed by [56].
Endoplasmic reticulum (ER): cellular organelle involved in the synthesis of lipids, proteins, and the regulation of calcium levels. Close physical contact sites between ER and mitochondria, known as MERCs, with biochemical isolated fractions called mitochondria-associated membranes (MAMs).
ER-associated degradation (ERAD) pathway: helps to ensure the quality control of newly synthesized proteins through the degradation of misfolded or unassembled proteins in the ER.
Intracristal space: region enclosed by cristae within the mitochondrial inner membrane, in which oxidative phosphorylation predominantly occurs.
Lipid droplets: organelles that hydrolyze and store neutral lipids.
Membrane fluidity: measure of microviscosity referring to the dynamic flexibility of lipid bilayers which is influenced by the collection of phospholipids contained, the nature of their attached fatty acids (length and saturation), and the incorporation of cholesterol and/or its derivatives.
MINFLUX: super-resolution microscopy technique that allows imaging with extremely high spatial resolution of proteins, including mitochondrial and cristae proteins [53].
Mitochondria: dynamic double-membraned cellular organelles with pluralistic roles extending to ATP synthesis, apoptosis, and calcium signaling.
Mitochondria–ER contact sites (MERCs): shorter than 50 nm between the ER and the outer mitochondrial membrane. Previously reviewed by [105].
Mitochondrial contact site and cristae organizing system (MICOS) complex: Consists of seven known protein subunits spanning the cristae junctions of the inner mitochondria membrane, with roles including cristae architecture remodeling. Previously reviewed by [60,61].
Mitochondrial fission 1 protein (FIS1): A mitochondrial outer membrane protein involved in mitochondrial fission through the recruitment of Drp1.
Mitochondrial fission factor (MFF): mitochondrial outer membrane protein involved in mitochondrial fission through the recruitment of Drp1. Aids in determining a fission type that is distinct from that of FIS1.
Mitochondrial permeability transitions (mPTs): events in which the inner mitochondrial membrane undergoes a sudden increase in permeability to ions and solutes, such as calcium, which can result in a loss of membrane potential and decreased ATP synthesis.
Mitophagy: selective degradation or removal of mitochondria through autophagy, often targeting damaged mitochondria.
mtDNA heteroplasmy: different sequences of mtDNA within the same individual, indicating mutations.
Nucleoids: dynamic structures composed of active mtDNA and nucleoid-associated proteins. Previously reviewed by [76].
OMA1: mitochondrial inner membrane protease involved in the regulation of mitochondrial dynamics through the proteolytic processing of OPA1.
Optic atrophy 1 (OPA1): mitochondrial inner membrane protein that is a principal regulator of mitochondrial fusion.
rRNA: structural and functional component of the ribosome, the cellular machinery responsible for protein synthesis. As with tRNAs and mRNAs, mitochondria have their own set of rRNA which is derived from mtDNA.
Ryanodine receptor 2 (RYR_2_): calcium-release channel primarily found in the sarcoplasmic reticulum of cardiac muscle cells, with mutations of RYR_2_ altering nanotunnel presence.
Transverse fusion–fission events: cristae fusion and fission events that happen across their inner boundary membrane.
X-type fusion–fission events: cristae merging and fission events that happen across their length, wherein adjoining cristae appear in an 'X' shape.
YME1L: mitochondrial inner membrane member of the AAA (ATPases associated with diverse cellular activities) family that proteolytically process OPA1 to modulate mitochondrial fusion.
Y-type fusion–fission events: cristae merging and fission events that happen across their length, wherein adjoining cristae appear in a 'Y' shape.
